# New Frontiers in Nutritional and Therapeutic Interventions for Obesity Phenotypes

**DOI:** 10.3390/medicina61040664

**Published:** 2025-04-03

**Authors:** Angelo Maria Patti, Rosaria Vincenza Giglio, Marcello Ciaccio, Anca Pantea Stoian, Teodor Salmen, Ioana-Cristina Bica, Imran Rangraze, Mohamed El Tanani, Manfredi Rizzo, Ali Abbas Rizvi

**Affiliations:** 1 Internal Medicine Unit, “Vittorio Emanuele II” Hospital, 91022 Castelvetrano, Italy; pattiangelomaria@gmail.com; 2 Department of Biomedicine, Neuroscience and Advanced Diagnostics, University of Palermo, 90133 Palermo, Italy; marcello.ciaccio@unipa.it; 3 Department of Laboratory Medicine, University Hospital, 90127 Palermo, Italy; 4 Department of Diabetes, Nutrition and Metabolic Diseases, Carol Davila University of Medicine and Pharmacy, 050474 Bucharest, Romania; ancastoian@yahoo.com; 5 Doctoral School, “Carol Davila” University of Medicine and Pharmacy, 050474 Bucharest, Romania; teodor.salmen@gmail.com (T.S.); cristina.ioanab@yahoo.com (I.-C.B.); 6 Internal Medicine Department, College of Medical Sciences, Ras Al Khaimah Medical & Health Sciences University (RAKMHSU), Ras Al Khaimah 11172, United Arab Emirates; imranrashid@rakmhsu.ac.ae (I.R.); eltanani@rakmhsu.ac.ae (M.E.T.); manfredi.rizzo@unipa.it (M.R.); 7 Department of Health Promotion Sciences Maternal and Infantile Care, Internal Medicine and Medical Specialties (Promise), University of Palermo, 90133 Palermo, Italy; 8 Unit of Diabetes and Cardiometabolic Prevention, University Hospital, 90127 Palermo, Italy; 9 Department of Medicine, Division of Endocrinology, Orlando VA Medical Center and University of Central Florida College of Medicine, Orlando, FL 32827, USA; aarizvi@aol.com

**Keywords:** obesity phenotypes, drugs, gut–brain axis, therapies, personalized medicine

## Abstract

The heterogeneity among patients with obesity is particularly evident in the weight loss response to interventions such as diets, drugs, devices and surgery. Obesity can be “catalogued” into four phenotypes: hungry brain (abnormal satiety for alteration of gut–brain axis), emotional hunger (hedonic eating), hungry gut (abnormal duration of satiety for faster gastric emptying) and slow burning (slowing of the metabolic rate). Phenotypes are grafted onto this complexity, the recognition of which allows for personalized medicine and increasingly targeted therapies. Although there are no standardized treatment protocols, we present management options consisting of lifestyle modifications and pharmacologic therapies. Nutritional advice and encouragement of adequate physical activity lead to increased self-efficacy and promote a sense of well-being when coupled with psychological approaches involving mindful eating. In summary, obesity has a complex pathophysiology best addressed through a therapeutic process suited to the phenotype encountered and in synergy with multifactorial interventions.

## 1. Introduction

Clinical obesity is a chronic and systemic disease characterized specifically by excess adiposity on physical examination and supported by laboratory or other diagnostic testing that reflect target organ dysfunctions. “Metabolically unhealthy” obesity is associated with a higher cardiometabolic risk [1].

Obesity, especially in its high-risk form, is a major driver of metabolic and cardiovascular alterations that can be mitigated by lifestyle modification and modified by pharmacotherapy [2]. The fight against obesity is undergoing major changes. The evolution of scientific knowledge regarding the etiology of obesity and its management has not led to a clear etiopathogenesis of the underlying causes that determine it [3]. The “obesity paradox” refers to an unexpectedly low prevalence of adverse health outcomes seen with higher body weight. It is, in part, caused by using Body Mass Index (BMI) as a measure of obesity rather than body fat distribution, exclusion of certain unmeasured cardiometabolic factors (e.g., cardiorespiratory fitness, sarcopenia, smoking) and bias due to disease-related weight loss [4].

The majority of cardiovascular disease (CVD) risk resulting from a high BMI or increased waist circumference is mediated by the presence of traditional risk factors such as atherogenic dyslipidemia, hypertension and diabetes mellitus [5]. In addition, individuals with obesity often develop cardiovascular disease at younger ages when they are apparently healthier and have fewer comorbid conditions than individuals with CVD who are not obese. When matched for BMI, the cardiometabolic risk profile of individuals with high visceral adipose tissue (VAT) accumulation had worse cardiometabolic risk profiles, increased risk of type 2 diabetes mellitus (T2DM) and more CVD outcomes compared to those with lower VAT [6].

For a long time, the pharmacological options for weight loss had been very few and of limited efficacy. The novel glucagon-like peptide-1 receptor agonist (GLP-1RA) drug class promises to radically change the landscape. These analogues of the intestinal hormone GLP-1, which include molecules such as liraglutide and semaglutide [7], along with the dual hormone analogue tirzepatide [8], are capable of inducing significant weight loss in a relatively short period of time. The weight loss is accompanied by marked benefits in the cardiovascular and metabolic parameters and a manageable side effect profile [8]. In this respect, these medications have been suggested as “game-changers”, since they are capable of significant and sustained weight loss when lifestyle interventions are not sufficient; further, these agents have several additional beneficial effects, reducing the risk of obesity-associated complications and ultimately improving patients’ quality of life [8].

A cornerstone in the management of obese patients is nutritional intervention. Nutritional therapy for obesity should not only be performed to reduce weight but also to improve health and quality of life. It encompasses increasing the intake of essential nutrients with low calorie content, choosing healthy foods such as fruits, vegetables, whole grains, lean proteins and healthy fats, avoiding processed foods rich in fat, sugar and salt, consuming foods rich in fiber or containing slow-absorbing starches and drinking plenty of water. An important consideration is the encouragement of a personalized meal plan based on the individual needs of the patient [9].

The aim of this review is to provide an overview of the nutritional and pharmacological therapies that could be implemented for the management of obesity, which manifests its heterogeneity in four phenotypes. Each obesity phenotype shows peculiar features and characteristics. For example, the hungry brain phenotype leads to a high intake of calories before reaching satiety, while the emotional hunger phenotype is characterized by a high level of anxiety, depression and emotional involvement in eating. The hungry gut phenotype refers to rapid gastric emptying, while the slow burning phenotype indicates a slowing of metabolism. To date, there has been no targeted and personalized intervention approach for each phenotype.

### 1.1. Research Strategy

We searched electronic scientific databases (i.e., MEDLINE (2000–2025), EMBASE and SCOPUS (2000–2025)), Web of Science Core Collection (since 2000) and available abstracts from national and international meetings. The leading search terms were clinical trials, obesity phenotypes, incretins, glucagon-like peptide-1 receptor antagonists, nutraceuticals and diet and their association with cardiovascular risk and prevention of CVD.

**Figure 1:**
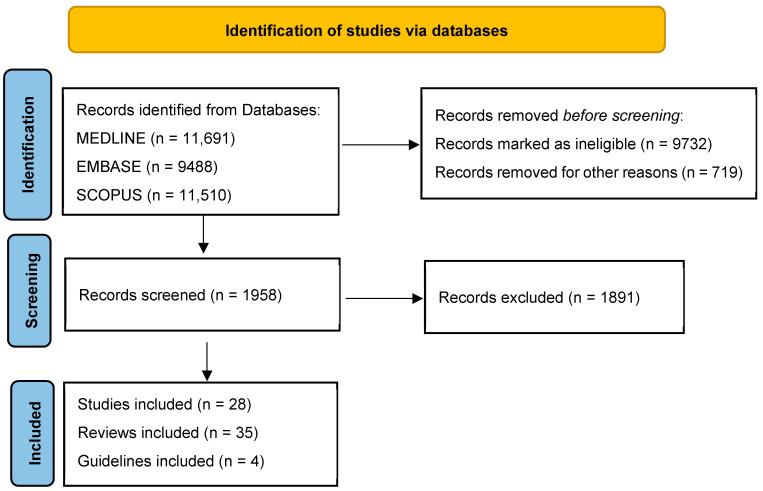
Flowchart of the research strategy.

Figure 1 summarizes the initial publication search performed by two authors and the number of papers from this search that are included in the review.

### 1.2. Obesity Phenotypes

Obesity is a form of malnutrition induced by a relative excess of calories and influenced by dietary, genetic, emotional and social factors. Most of the association between adiposity and CVD is explained by altered cardiometabolic risk factors in the setting of “high-risk obesity” [10]. In contrast to the latter, the term “metabolically healthy obesity” was coined to refer to a subgroup of low-risk individuals who, despite being obese, have low levels of VAT, generally follow a healthy diet, and are physically active [11]. Moderate-risk obesity is characterized by visceral obesity and characteristics of metabolic syndrome (MetS), which puts these individuals at high risk of cardiovascular events, particularly if they are sedentary and have inadequate eating habits [12].

There appears to be a close relationship between the digestive system and the central nervous system, and the balance of this communication pathway is impacted by numerous factors [13]. The above risk-based profile highlights that, in reality, obesity cannot be classified solely from the point of view of anthropometric parameters, because they are very variable and lack unequivocal interpretation, favoring the approach of a tailor-made therapy for these subjects [14]. The current tendency is to classify obesity into for phenotypes based on the characteristics of an organism that result from the interaction between its genetic constitution and the environment [15]. Figure 1 represents the different ways in which an individual can accumulate body fat and the targeted therapeutic strategies effective in reducing body fat so that the individual has a weight considered normal and metabolically protective. The first phenotype is the so-called “hungry brain”, mainly controlled by the gut–brain axis, in which the subject requires a large number of calories to achieve fullness and satiety. The second phenotype is the “hungry intestine”, characterized by an abnormally long duration of fullness, with relatively rapid gastric emptying. The third phenotype signifies “emotional hunger”, or the desire to eat to cope with positive or negative emotions, defined as hedonic behavior. Finally, the fourth phenotype is termed “slow combustion”, which is the state of the slowing down of the metabolic rate [16]. The “hungry brain” phenotype requires a high calorie intake before reaching fullness and satiety; the “emotional hunger” phenotype is associated with higher levels of anxiety, depression and emotionally driven eating, as well as lower levels of self-esteem and a worse body image; the “hungry intestine” phenotype shows more accelerated gastric emptying by feeding more often; and the “slow combustion” phenotype, with a slowed metabolism, shows lower muscle mass and a lower predisposition to physical activity [16].

**Figure 2:**
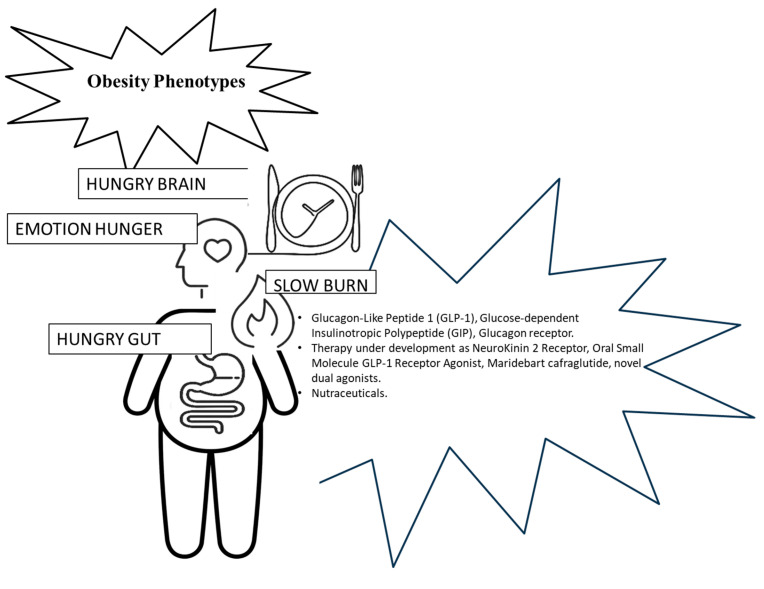
Obesity phenotypes and treatments.

The premise of this review is that the identification of biologic patterns is important for a personalized therapeutic approach. Although there is no uniformity of management of any single phenotype, certain strategies can be identified to manage them. Figure 2 gives a representative guideline that could help to better understand the available treatment options, keeping in mind that there is a lack of current standardized protocols based on the type of obesity.

## 2. Management of Obesity

### 2.1. Drugs

The newer drugs indicated for obesity were originally conceptualized as therapies for T2DM. They are analogues of GLP-1 and are capable of activating the same receptors as the native hormone [17]. GLP-1 is produced and released by the intestine after a meal and acts on receptors in the pancreas, liver, muscle and brain, increasing glucose-dependent insulin secretion and inhibiting glucagon secretion by modulating pancreatic beta and alpha cells, respectively. It also increases satiety and slows gastric emptying [17]. In this way, by mimicking its effects, the GLP-1RA can reduce blood glucose levels, with side effects comparable to traditional anti-diabetic drugs [18]. However, they also have a beneficial secondary effect of impressive weight loss, leading to their testing in the field of obesity [19]. The weight reduction is mediated by the action that these drugs have on the central nervous system, on the neurons that regulate the sense of hunger and satiety, and the desire for food [20]. Therefore, these drugs essentially increase the sense of fullness and reduce the amount of food consumption.

There are currently two members of the GLP-1RA class approved for obesity, liraglutide and semaglutide. A third agent, tirzepatide, is a novel dual-receptor agonist of both GLP-1 and Glucose-dependent Insulinotropic Polypeptide (GIP). As they are peptides that are subject to gastric degradation, these agents cannot be taken orally and have to be injected subcutaneously [21]. Liraglutide is a first-generation daily drug with encouraging efficacy data showing an average weight loss of around 8–10% of the initial weight [22]. The second-generation agent semaglutide, and even more so the “twincretin” tirzepatide, have an even higher efficacy; the former induces around a 16% weight loss, while it approaches 20–22% with the latter [23]. It should be kept in mind that these are average weight loss percentages, and results vary in different individuals. Although the average duration of follow-up in most trials is usually one year, efficacy data of between two and four years confirm that the results can be maintained even in the long term by regular adherence to these medications. Even though these drugs are highly efficacious, in most patients, suspending therapy inevitably leads to weight regain. This is an entirely expected phenomenon, because obesity is a chronic disease that requires long-term treatment [24].

We must abandon the concept that achieving weight loss by any means is the solution to the obesity problem. Patients can lose weight in many different ways, including nutritional modification [25]; however, if the intervention is not maintained over time, the weight is likely to be regained. The most common side effects of these drugs affect the gastrointestinal system and, although frequent, they can be managed by modulating the therapy to a to a lower dose. They tend to attenuate overtime; for some people they may be excessive, but they do not represent a long-term problem for most patients [26]. Probably the most suitable candidates for these drugs are those with moderate to severe obesity and who have metabolic and cardiovascular complications; for these individuals, losing 10–15 kg would result in significant clinical benefits [27].

Although there is insufficient data in the extremely severe category of obesity, it is likely that the weight loss that these drugs can produce is variable. These degrees of obesity necessitate bariatric surgery, which can be accompanied by pharmacological therapy, both before and after the surgery [28].

The SCALE study involved a total of 3731 non-diabetic obese patients who were using liraglutide administered in a weekly subcutaneous dose of 3 mg for 56 weeks [29]. These patients were randomly assigned to receive either placebo (n = 1244) or liraglutide (n = 2487) in conjunction with lifestyle intervention. The study showed that 63.2% of patients who received liraglutide had a weight loss of approximately 5% of their body weight, compared to 27.1% in the placebo group. In addition, 33.1% of patients treated with liraglutide achieved a weight loss of approximately 10% of their initial body weight, while only 10.6% of those treated with placebo achieved the same result [29].

Semaglutide at a dose of 2.4 mg results in reduced appetite, increased fullness and satiety and improved eating behavior [30]. In clinical trials, 56 weeks of semaglutide therapy in combination with a 500 kcal/day diet resulted in 14.9% weight loss versus 2.4% with placebo in people without diabetes [31]. When compared with 3 mg liraglutide, 2.4 mg semaglutide demonstrated twice the weight loss [32]. Weight loss with semaglutide was associated with improvements in quality of life and physical function [33]. In addition to the improvement in multiple cardiovascular risk factors with 2.4 mg semaglutide, the cardiovascular safety profile was also confirmed in people with obesity. In the SUSTAIN 6 study, 1 mg semaglutide in subjects with T2DM and established CVD resulted in a reassuring 26% reduction in major adverse cardiac events (MACE) compared with placebo, primarily due to a reduction in the incidence of stroke [34].

Obesity induces a pro-inflammatory state that can contribute to endothelial dysfunction and coronary microvascular impairment [35], finally resulting in heart failure with preserved ejection fraction (HFpEF); there is increasing clinical evidence supporting the role of GLP-1 receptor agonists in patients with obesity-related HfpEF [36]. The impact of 2.4 mg semaglutide in people with HFpEF and obesity is being evaluated in the ongoing STEP-HFpEF and STEP-HFpEF DM studies [36]. Oral semaglutide, an approved treatment for type 2 diabetes at doses of 7 and 14 mg once daily, is currently undergoing phase 3 studies as a treatment for obesity at a dose of 50 mg once daily. In a phase 2 dose-finding study, 40 mg of oral semaglutide in individuals with T2DM resulted in a weight loss of 6.9% compared with 6.4% with 1 mg injectable semaglutide and 1.2% with placebo [37].

Tirzepatide is the first—and to date the only—treatment belonging to a new therapeutic class that activates both the GIP and GLP-1 hormone receptors [38]. By binding both receptors, the drug increases pancreatic insulin secretion, enhances insulin sensitivity and reduces food intake. The half-life is approximately 5 days, and it is administered subcutaneously once a week. What differentiates tirzepatide is the GIP receptor agonism, which acts on mechanisms related to weight and has beneficial actions at the level of adipose tissue, caloric intake and the reduced feeling of nausea [38].

The efficacy of tirzepatide in weight management is based on robust clinical trial evidence. The key trials include the phase III SURMOUNT-1 study, conducted in adults with obesity or overweight with at least one weight-related comorbidity but without T2DM, and the SURPASS clinical trials, evaluating participants with obesity or overweight and T2DM [39]. Different doses of tirzepatide (5 mg, 10 mg and 15 mg) have all shown significant weight reduction in obese patients with T2DM compared to 1 mg semaglutide, with the average weight loss ranging from 7.6 kg to 12.9 kg [40]. In the phase III SURMOUNT-1 clinical trial, tirzepatide, at a maintenance dose of 5 mg reached in 4 weeks, when used with diet and exercise, demonstrated a weight reduction of 16% at week 72 [41]. With a maximum maintenance dose of 15 mg, the average weight loss to date has been 22.5% [40]. In summary, all five global pivotal studies of the SURPASS program have demonstrated a reduction in glycated hemoglobin and body weight. The most common side effects were gastrointestinal, such as nausea and diarrhea; in general, they were mostly mild or moderate in severity, occurred most often during dose escalation and decreased over time.

### 2.2. Obesity Therapies Under Development

Although GLP-1-based therapies have revolutionized the care of patients with obesity and T2DM, new strategies need to be developed that exploit the body’s ability to burn calories while reducing appetite. There are three members of the GLP-1RA class currently available, but the panorama is destined to quickly expand in the coming years. Combinations of multiple molecules are being developed that mimic the effect of a variety of hormones for enhanced efficacy. An example is the combination of semaglutide and cagrilintide [42]; the latter is an analogue of amylin, a hormone that regulates eating behavior. Triple-agonists, such as retatrutide, which is an agonist of GLP-1, GIP and glucagon, and survodutide, which is a dual agonist of GLP-1 and glucagon, are in the advanced phase of development [42].

In addition to new targets, agents with different mechanisms of action are also expected to be developed. With this expanding armamentarium of available drugs, our ability to personalize therapy and find the most suitable agent for individual patients will increase, representing a further benefit. Some of these molecules could prove more effective for weight loss, while others could carry a more favorable side-effect profile; still others, perhaps, could be more effective for certain types of patients. Orforglipron, for example, is a non-peptide analogue of GLP-1 in an advanced stage of development. As a result of its novel structure, it is able to withstand gastric passage and can be administered orally with good efficacy [43].

Action on receptors other than GLP1/GIP are potential avenues of investigation. Bimagrumab is a monoclonal antibody with a less pronounced action on weight than the GLP-1 analogues but with the unique property of preserving lean muscle mass [44]. Drug treatments that target NeuroKinin 2 Receptor (NK2R) could be a viable option for fighting obesity by reducing hunger, burning energy, and improving insulin resistance, without causing unwanted side effects [45]. Activation of the NK2R has been shown to improve the utilization of fuel and reduce appetite without causing nausea. It also reduces body weight, ameliorates hyperglycemia and improves the lipid profile by increasing insulin sensitivity [45].

The results from a Phase I clinical trial for CT-996, an oral small molecule GLP-1 receptor agonist designed to treat obesity and T2DM, showed a mean placebo-adjusted weight loss of 6.1% over four weeks [46]. CT-996 is specifically designed to be a biased GLP-1 receptor agonist that activates cyclic Adenosine 3′,5′ MonoPhosphate (cAMP) signaling with little or no recruitment of beta-arrestin [46]. The results also indicated that the treatment did little to change blood chemistry, either fasting or after a standardized high-fat meal, suggesting that it could be dosed regardless of meal timing. Thus, CT-996 could not only aid in glycemic control and weight loss but could potentially serve as maintenance therapy after injectable-induced weight loss.

VK2735 is a new anti-obesity drug that mimics the action of both GLP-1 and GIP [47]. In the Multiple Ascending Dose (MAD) study, a phase 1 clinical trial, the drug produced dose-dependent weight reductions of up to 8.2% more compared to baseline and 6.8% more versus placebo in 28 days at a maximum dose of 100 mg daily. Up to 100% of patients treated with VK2735 achieved a weight loss of 5% or greater, and durability of weight loss was observed with an 8.3% reduction extending to the 57th day of follow-up—4 weeks after the administration of the last dose. Based on a preliminary evaluation of the weight loss trajectories at the different doses, continued treatment beyond 28 days predicted further weight reduction. In a MAD study update, the safety and tolerability profile of VK2735 in pill form was confirmed over 28 days at a daily dose of 100 mg [47].

MariTide (Maridebart cafraglutide-AMG133), administered once a month, targets GLP-1 and GIP; however, instead of stimulating GIP receptors, it blocks them. Subjects who took MariTide also lost more than 14% of their body weight in 12 weeks. It is not entirely clear why both stimulation and blockade of these receptors appear to promote weight loss [48]; research shows that mice and humans with GIP receptor mutations weigh less.

Finally, as mentioned previously, the triple-receptor-agonist retatrutide works by targeting receptors for GLP-1, GIP and glucagon, a hormone that helps to burn fat. Data from a trial published recently showed that retatrutide helped people lose more than 17 percent of their body weight after 24 weeks. By 48 weeks after treatment initiation, participants had lost an average of 24 percent of their weight, more than any other drug on the market [28].

### 2.3. Cardiovascular Outcome Trials (CVOTs) in Obese Patients

Clinicians should partner with patients in promoting obesity screening and encourage weight loss to improve their quality of life. Health efforts should prioritize primary prevention and obesity management to reduce the risk of CVD and related complications [48]. In its new consensus statement, the European Society of Cardiology (ESC) recommends weight loss and physical exercise to improve metabolic control and reduce the risk of CVD in overweight or obese diabetic patients. Exercise training performed at maximal fat oxidation (FATmax) is an efficient non-pharmacological approach for the management of obesity and related cardiometabolic disorders. Relative heart rate rather than relative oxygen uptake should be used to establish FATmax reference values in obese patients. The volume of training must be higher in adults to achieve similar fat oxidation compared to adolescents, while treadmill exercise requires a lower training volume to achieve significant fat oxidation compared to stationary cycling [49].

In terms of parameters, an optimal BMI between 20 and 25 kg/m^2^ and a waist circumference of less than 94 cm for men and 80 cm for women are recommended [4].

Obese or pre-diabetic patients should receive antihypertensive drugs if their blood pressure is equal to or greater than 140/90 mmHg, or 130–139/80–89 mmHg if they already have risk factors or their lifestyle is not sufficient to lower their weight/height ratio [50]. For weight loss in overweight or obese patients, drugs such as GLP-1RAs with proven cardiovascular effects are preferred [51,52].

The SELECT (Semaglutide and Cardiovascular Outcomes in Obesity without Diabetes) study demonstrated that semaglutide, administered subcutaneously at a dosage of 2.4 mg once a week, reduces cardiovascular risk in overweight and obese subjects as secondary prevention [53]. The multicenter, double-blind, randomized trial enrolled approximately 17,000 male and female patients aged 45 years or older with a BMI of 27 kg/m^2^ or more, a history of at least one cardiovascular event (myocardial infarction, ischemic stroke, or lower limb arterial disease) and no diabetes. The study compared weekly dosing of 2.4 mg semaglutide with placebo as an add-on to standard care for the prevention of MACE over five years. It achieved its primary objective by demonstrating a statistically significant 20% reduction in MACE for people treated with 2.4 mg semaglutide compared to placebo. The primary endpoint of the study was defined as the composite outcome of the first occurrence of MACE, defined as cardiovascular death, non-fatal myocardial infarction or non-fatal stroke. This study was the first and only one to have demonstrated that a drug used for the treatment of obesity reduced the risk of cardiovascular events. In particular, a greater benefit was demonstrated in patients with a lower BMI: weight loss equal to kg −9.39 ± 0.09%; waist circumference −7.56 ± 0.09%; glycated hemoglobin level −0.31 ± 0.00%; systolic blood pressure mmHg −3.82 ± 0.16%; diastolic blood pressure mmHg −1.02 ± 0.10%; heart rate—beats/min 3.79 ± 0.11; high-sensitivity CRP level −39.12%; total cholesterol level −4.63%; LDL cholesterol level −5.25%; triglyceride level −18.34%. Early use of the drug could therefore have significant implications [53].

The SUMMIT (Study of Tirzepatide in Participants With Heart Failure With Preserved Ejection Fraction and Obesity) study is a CVOT involving tirzepatide. This study aims to recruit 700 participants with obesity and a diagnosis of heart failure with preserved ejection fraction. Participants were randomized to tirzepatide or placebo. The primary outcome is a hierarchical composite of all-cause mortality, heart failure events, 6 min walk distance and Kansas City Cardiomyopathy Questionnaire Clinical Summary Score category. The study was completed in July 2024. The study showed that patients treated with tirzepatide showed a lower percentage of heart failure (9.8% vs. 15.3%, *p* = 0.026) and confirmed death from cardiovascular causes (2.2% vs. 1.4%, *p* < 0.001) than the placebo group [54].

Finally, in the SURMOUNT-MMO (A Study of Tirzepatide on the Reduction on Morbidity and Mortality in Adults With Obesity) study, 15,000 obese participants with either established CVD or at risk for it will be randomized to either tirzepatide or placebo and followed for five years [55]. Currently in the recruitment phase, the primary outcome of the study is the occurrence of any component of the composite CV outcome.

### 2.4. Diet Composition and Nutraceuticals

Carbohydrates, proteins and fats are the main types of macronutrients, defined as nutrients that are needed daily in large amounts. They provide 90% of the dry weight of the diet and 100% of the energy. All three provide energy (measured in calories). These nutrients are also distinguished by the speed with which they provide energy: carbohydrates act the fastest and fats the slowest. The body uses these building blocks to form the substances it needs for growth, survival and movement (including other carbohydrates, proteins and fats). The glycemic index ranks foods based on how quickly the carbohydrates they contain raise blood sugar levels. The values range from 1 (the slowest) to 100 (the fastest, the pure glucose index). However, the actual rate at which the level rises also depends on what other foods are eaten at the same time and other factors. The glycemic index tends to be lower for complex carbohydrates than for simple carbohydrates [56].

For a given number of calories ingested, decreasing the carbohydrate intake (low-carb diet <40%) amplifies weight loss and reduces the degree of hepatic steatosis [57]. Among very-low-calorie diets (low-carb diet <40%) and restricted carbohydrate intake, ketogenic ones (<50 g of carbohydrates/day) are particularly effective in reducing body weight and hepatic steatosis [58]. Foods with a low glycemic index are preferred because they stimulate insulin secretion to a lower degree, inhibiting its antilipolytic and lipogenic action. These include apples, cherries, strawberries, citrus fruits, carrots, dried legumes, fish, milk and yogurt. On the other hand, foods with a high glycemic index such as sugar, sweets, ice cream, jam, honey, figs, lotuses, bananas, grapes, sugary drinks, white bread, white rice, potatoes, pizza, rusks, breadsticks, cornflakes, crackers and popcorn, must be limited [56,59].

The amount of dietary fiber is very important, as a high intake reduces the glycemic index of meals, increases the feeling of satiety and reduces overall calorie intake. Therefore, priority should be given to foods rich in fiber, such as fruits (no more than three portions per day), vegetables, legumes and whole grains. An increase in the intake of polyunsaturated fatty acids is associated with a reduction in body and liver fat and an increase in lean mass, while an increase in saturated fatty acids has the opposite effect [60]. Increased consumption of nuts, which are rich in both unsaturated fats and dietary fiber, is associated with a reduction in weight and body fat; the high fiber content is thought to reduce the absorption of fat [61]. From a practical standpoint, the recommended foods to limit saturated fat and maintain a low glycemic index include turkey breast, chicken breast, lean beef, egg whites, yogurt, kefir, mozzarella and legumes.

Increased dietary protein intake promotes both the induction and maintenance of weight loss. Proteins increase muscle mass, which plays an important role in energy expenditure, the retardation of stomach emptying and an increase in the sense of satiety. They inhibit the sense of hunger at the level of the hypothalamic centers that control food intake and satiety [62,63]. The recommended daily protein intake for the promotion of weight loss is 1.2–1.4 g per kg of ideal body weight. Furthermore, protein enrichment improves stabilization after weight loss compared to other dietary strategies (DIOGENES study) [64].

Obesity is associated with low-grade inflammation that is worsened by red and processed meat, white bread and fried, processed and ultra-processed foods such as bacon, frankfurters, chips, snacks, milk chocolate and sugary drinks. Obesity stimulates some inflammatory pathways without triggering a frank, serious and acute picture of inflammation. There are numerous foods that help the body strengthen its immune defenses, thus preventing inflammatory conditions and encouraging their treatment. These foods, rich in nutrients with inflammatory properties, have antioxidant properties and are rich in omega 3 fatty acids and vitamins, especially vitamins C and D. The essential principle of the anti-inflammatory diet is to increase the molecules that defend us from oxidative stress and can positively affect systemic inflammation. Anti-inflammatory foods and drinks include oily fish, berries, cherries, broccoli, avocado, peppers, mushrooms, turmeric, tomatoes, dark chocolate and green tea [65,66].

**Figure 3:**
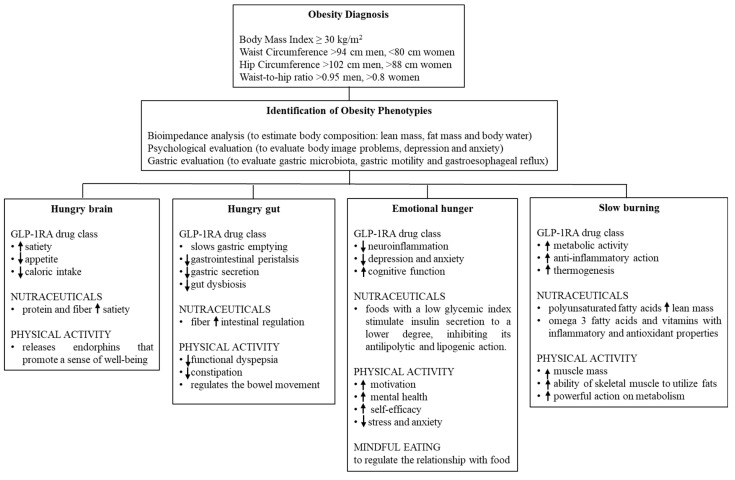
Hypothetical management approach for the 4 obesity phenotypes.

A dietary strategy that has received a lot of interest is intermittent fasting. Although it has various models, at the moment it is not possible to define whether any of them (time-restricted eating, alternate-day fasting and the 5 diet) have any advantages over the common low-calorie diet regarding weight loss or cardiometabolic risk [67]. There are currently no standardized dietary protocols to apply to different obesity phenotypes. Figure 3 shows hypothetical foods and the effects they exhibit on several phenotypes.

## 3. Conclusions

Great strides have been made towards knowledge related to the definition, pathophysiology and management of obesity. Obesity is a chronic and systemic disease that may present a varying degree of cardiometabolic risk. However, the availability of this information has had a limited translational impact on disease management worldwide.

An increased awareness of obesity phenotypes could facilitate individualized and “precision” medicine and promote tailored management. These phenotypes are somewhat distinct physiologically; the only phenotype that requires a psychological approach is emotional hunger. Each phenotype has well-defined characteristics that can be managed through pharmacological, nutritional and lifestyle strategies.

Pharmaceutical innovations appear promising in the fight against obesity and related comorbid conditions; these new therapies must be administered in order to act on the alterations that caused weight gain (reduced satiety, increased caloric intake, increased dysbiosis, increased depression, reduced metabolic activity). The same applies to physical activity aimed at promoting a sense of well-being, promoting intestinal transit, improving self-esteem and enhancing muscle mass. There are multiple studies showing that appropriate dietary choices regarding fiber, protein and unsaturated fat intake can promote weight loss and general health in the various phenotypes.

Obesity and its consequences require urgent and incisive interventions to counteract their impact on societal health. Intersectoral policies are needed that consider all the sociocultural, environmental, relational and emotional determinants that influence eating habits and lifestyle, with the aim of creating the conditions that encourage nutritionally optimal food choices and promote an active lifestyle.

## Abbreviations

The following abbreviations are used in this manuscript:

BMI	Body Mass Index
cAMP	Cyclic Adenosine 3′,5′ Monophosphate
CVD	Cardiovascular Disease
ESC	European Society of Cardiology
FATmax	Maximal Fat Oxidation
GIP	Glucose-Dependent Insulinotropic Polypeptide
GLP-1RA	Glucagon-Like Peptide-1 receptor agonist
HFpEF	Heart Failure with Preserved Ejection Fraction
MACE	Major Adverse Cardiac Events
MAD	Multiple Ascending Dose
MetS	Metabolic Syndrome
NK2R	Neurokinin 2 Receptor
T2DM	Type 2 Diabetes Mellitus
VAT	Visceral Adipose Tissue
